# IRMA: Machine learning-based harmonization of $^{18}$F-FDG PET brain scans in multi-center studies

**DOI:** 10.1007/s00259-025-07114-4

**Published:** 2025-02-18

**Authors:** S.S. Lövdal, R. van Veen, G. Carli, R. J. Renken, T. Shiner, N. Bregman, R. Orad, D. Arnaldi, B. Orso, S. Morbelli, P. Mattioli, K. L. Leenders, R. Dierckx, S. K. Meles, M. Biehl, for the Alzheimer’s Disease Neuroimaging Initiative

**Affiliations:** 1 Department of Nuclear Medicine and Molecular Imaging, University Medical Center Groningen, Groningen, Netherlands; 2 Bernoulli Institute for Mathematics, Computer Science and Artificial Intelligence, University of Groningen, Groningen, Netherlands; 3 Department of Neurology, University of Michigan, Ann Arbor, MI USA; 4 Department of Biomedical Sciences of Cells & Systems, Cognitive Neuroscience Center, University Medical Center Groningen, Groningen, Netherlands; 5 Cognitive Neurology Unit, Neurological Institute, Tel Aviv Sourasky Medical Center, Tel Aviv, Israel; 6 Faculty of Medicine and Health Sciences, Tel Aviv University, Tel Aviv, Israel; 7 Sagol School of Neuroscience, Tel Aviv University, Tel Aviv, Israel; 8 Department of Neuroscience, Rehabilitation, Ophthalmology, Genetics, Maternal and Child Health (DINOGMI), University of Genoa, Genoa, Italy; 9 Neurophysiopathology Unit, IRCCS Ospedale Policlinico S. Martino, Genoa, Italy; 10 Nuclear Medicine Unit, IRCCS Ospedale Policlinico S. Martino, Genoa, Italy; 11 Department of Neurology, University Medical Center Groningen, Groningen, Netherlands; 12 SMQB, Institute of Metabolism and Systems Research, College of Medical and Dental Sciences, University of Birmingham, Birmingham, UK

**Keywords:** $^{18}$F-FDG PET, Neuroimaging, Site effect, Harmonization, Machine learning, Neurodegeneration

## Abstract

**Purpose:**

Center-specific effects in PET brain scans arise due to differences in technical and procedural aspects. This restricts the merging of data between centers and introduces source-specific bias.

**Methods:**

We demonstrate the use of the recently proposed machine learning method Iterated Relevance Matrix Analysis (IRMA) for harmonization of center-specific effects in brain $^{18}$F-Fluorodeoxyglucose ($^{18}$F-FDG) PET scans. The center difference is learned by applying IRMA on PCA-based feature vectors of healthy controls (HC), resulting in a subspace $\varvec{V}$, representing information not comparable between centers, and the remaining subspace $\varvec{U}$, where no center differences are present. In this proof-of-concept study, we demonstrate the properties of the method using data from four centers. After center-harmonization, a Generalized Matrix Learning Vector Quantization (GMLVQ) model was trained to discriminate between Parkinson’s disease, Alzheimer’s disease and Dementia with Lewy Bodies.

**Results:**

At the initial IRMA iteration, the system was able to determine the center origin of the four HC cohorts almost perfectly. The method required six iterations, corresponding to a six-dimensional subspace $\varvec{V}$, to determine the entire center difference. An uncorrected disease classification model was highly biased to center-specific effects, creating a falsely inflated performance when applying internal (cross-) validation. The cross-validation performance of the center-harmonized model remained high, while it generalized significantly better to unseen test cohorts. Furthermore, the framework is highly transparent, providing analytic reconstructions of the correction and visualizations of the data in voxel space.

**Conclusion:**

IRMA can be used to learn and disregard center-specific information in features extracted from brain $^{18}$F-FDG PET scans, while retaining disease-specific information.

**Supplementary Information:**

The online version contains supplementary material available at 10.1007/s00259-025-07114-4.

## Introduction

Molecular neuroimaging in the study of neurodegenerative disorders has advanced significantly in recent years and is currently facing complex challenges such as the validation of biomarkers for differential diagnosis, prediction and prognosis, therapy stratification and follow-up [1]. Addressing these questions necessitates access to extensive patient cohorts and consequently multi-center collaborations are increasingly common for the aggregation of larger data sets. However, differences in technical and clinical acquisition protocols give rise to center-specific effects in the resulting images. This is a major issue for direct comparability between cohorts, especially when pooling data retrospectively. This paper addresses the harmonization of features extracted from multi-center brain PET data, to facilitate large-scale retrospective studies in molecular neuroimaging.

Brain imaging with $^{18}$F-Fluorodeoxyglucose Positron Emission Tomography ($^{18}$F-FDG PET) offers insights into the regional cerebral glucose metabolism, reflecting underlying changes in neuronal activity in response to a neurodegenerative process. In clinical practice, $^{18}$F-FDG PET is used to distinguish between a variety of neurodegenerative conditions [2]. Each condition has a characteristic pattern of cerebral hypometabolism, which can be visually evaluated by an expert PET reader. For instance, in patients with dementia, a diagnosis of Alzheimer’s disease (AD) is supported by hypometabolism in the posterior cingulate cortex and precuneus, with sparing of the occipital cortex. In contrast, in patients with dementia with Lewy bodies (DLB), the occipital cortex is typically affected [3]. $^{18}$F-FDG PET is also routinely used in the work-up of patients with movement disorders, to distinguish between Parkinson’s disease (PD) and atypical parkinsonism [4]. In a research setting, proposed biological classification schemes also include $^{18}$F-FDG PET as a biomarker of neuronal degeneration. These include the ATN classification scheme for AD, as well as the SynNeurGe diagnostic criteria for PD [5, 6]. Classification of $^{18}$F-FDG PET scans using machine learning is especially promising for the differential diagnosis of neurodegenerative diseases, due to its natural ability to model multiple target variables (classes) simultaneously, and data-driven modeling of complex relationships in high dimension [7–10].

Constructing an $^{18}$F-FDG PET image involves multiple variables, ranging from tracer dose and scanner model to acquisition time and reconstruction settings. Therefore, obtaining identical protocols between medical centers is a difficult problem. Differences in these protocols give rise to center-specific effects, for example in resolution, signal-to-noise ratio, and count rate, but also topographically, for example due to differences in implementations of point spread function (PSF) and scatter correction [11–16]. In this work, we refer to the protocol-specific effects as center effects, assuming that the center did not change their protocol while obtaining scans for the considered cohort. Overall, absolute PET quantification remains challenging [17]. Even though quantification would be performed according to gold standard by including arterial blood sampling, center differences would still remain due to the additional layer of quantification introduced by the reconstruction process. For brain $^{18}$F-FDG PET, researchers typically use semi-quantification by applying global mean or reference region-based normalization techniques [18]. Independently of the semi-quantification method used, limitations in direct comparability between images produced by different reconstruction procedures and scanners introduces major difficulty for external (cross-center) validation of any statistical or machine learning-based model which relies on uniform representation of the PET images [11, 19].

Multiple efforts for multi-center harmonization in neuroimaging have been made. One possibility is prospective image-based harmonization, promoted by the European Association of Nuclear Medicine via the EARL standards [20, 21]. For brain PET/CT [22, 23], this involves recommended reconstruction settings to achieve a minimum image quality in a Hoffman 3D brain phantom [24], and using the Hoffman phantom to estimate a smoothing kernel which obtains comparable resolutions between centers [23]. While high-quality images and improved prospective harmonization is always to be encouraged, it is not always feasible, especially for retrospective image studies. Additionally, the EARL guidelines do not address potential differences in the topography, or differences that may only become apparent in a derived feature space.

Feature-based harmonization is an alternative approach. The ComBat method was first introduced for genomics [25] and later applied to radiomics and neuroimaging [26–30]. In ComBat, features extracted from an image are harmonized based on an empirical Bayes model, assuming that the center effect can be modeled by an offset and a multiplicative coefficient. The method is typically applied to feature distributions in patient populations, indirectly aligning the corresponding means and standard deviations.

While the ComBat method is commonly used in radiomics in oncology, applying ComBat on features from patients with neurodegenerative diseases is likely to be problematic, due to the large heterogeneity observed in the neurodegenerative spectrum [31, 32]. Thus, it is rarely reasonable to assume that patient cohorts should have similar distributions, even in cohorts with the same disease duration. Additionally, when it comes to machine learning applications, a large number of features are typically considered, and sample sizes required for an accurate statistical estimation increase steeply with the number of parameters or dimensions [33]. This is an issue in fields such as clinical neuroimaging, where the size of available cohorts is typically limited. If the parameters are not estimated accurately, the harmonization procedure might even introduce new bias in the data [34]. In general, harmonization methods for brain $^{18}$F-FDG PET in the context of neurodegeneration have not been widely studied.

Subspace correction was introduced by van Veen et al. as a machine learning method for removing unwanted variance [35] from feature vectors. The procedure was applied for the removal of center-specific effects in $^{18}$F-FDG PET scans, where features had been extracted using principal component analysis (PCA). The method involves training a Generalized Matrix Learning Vector Quantization (GMLVQ) model to discriminate between healthy controls (HC) from different centers. This results in a single vector in binary problems, or a set of vectors (i.e. a subspace) in the multi-class case, which enables a clear separation between the centers. Once the center differences are identified and represented mathematically, they can be projected out, and hence removed from consideration. Thereafter, a corrected model can be trained on a disease classification problem. The results showed that the method removed a meaningful part of the center-specific variance present in the $^{18}$F-FDG PET scans. This enabled the reduction of bias in the disease classification model and a more meaningful interpretation of feature relevances. However, due to the single application of the procedure it was likely that residual unwanted center-specifics were still exploited by the classifier.

In this work, we apply an iterative subspace correction procedure, called Iterated Relevance Matrix Analysis (IRMA). The method involves training a GMLVQ model to classify the center origin of HC cohorts, like in the work by van Veen et al. [35], but iteratively accumulating center-discriminative vectors and retraining the model while projecting out all previously found discriminative directions. This is continued until no further meaningful separation can be found between the HC groups of different origin. IRMA was recently introduced as a means to improve the interpretation of feature relevances in classification problems, and for extracting class-discriminative subspaces [36, 37]. In this work, we apply and validate the method for the removal of center-specific effects in $^{18}$F-FDG PET brain scans, and show that the iterative procedure is necessary to extract *all* center-specific information, as opposed to the single step applied by van Veen et al [35].

Using the basic assumption that age- and gender-matched HC cohorts from different centers should have similar distributions in feature space, we present the application of IRMA in a four-center setting, including HCs and three disease classes (AD, PD and DLB). First, we investigated whether the method could remove center-specific information in PCA-based features, such that the four HC cohorts were indistinguishable after the correction. Second, we evaluated the impact of the correction when training a model to classify the three disease classes, with each group in the training cohort scanned at a different center, and estimated the level of bias in an uncorrected model. We used unseen test cohorts to estimate cross-center classification performance before and after harmonization, and compared our results to two alternative harmonization methods: center-wise z-scoring and ComBat. While IRMA requires HC cohorts for the harmonization, the particular diseases included in this work (AD, PD, and DLB) represent an example of a classification problem relevant to the research field of neurodegenerative diseases [38], which can be addressed using the proposed harmonization method. However, IRMA could potentially be applied to any radiotracer or imaging modality, and any condition of interest. Finally, we present analytical expressions for representing the images in 3D voxel space before and after correction.

## Material and methods

Here, we cover the background of the machine learning algorithms in this work, and the experimental setup. This involves the GMLVQ framework in the “GMLVQ” section, and the IRMA procedure in the “IRMA” section. Furthermore, we cover our patient cohorts in “Patients”, image processing pipelines in “[Sec Sec6]”, and finally equations for quantifying the correction in voxel space together with the experimental setup in “Experimental setup”.

### GMLVQ

Generalized Matrix Learning Vector Quantization (GMLVQ) belongs to the family of prototype-based classifiers, where the basic form of of LVQ was introduced by Kohonen in 1986 [39, 40]. In LVQ, data points $\textbf{x} \in \mathbb {R}^N$ are assigned to one of *C* classes based on the label of the nearest prototype. A prototype is a vector $\textbf{w}_j \in \mathbb {R}^N$ which serves as a typical representative of a class. In the following, we assume that each class is represented by exactly one prototype, i.e. $j\in \left\{ 1,2,\ldots , C\right\} .$

Details of the update procedure depend on the flavor of LVQ [41, 42]. Sato and Yamada introduced Generalized LVQ (GLVQ), formulating the training in terms of a cost function, which enables updates of the model by gradient descent [43]. Simply put, the objective is placing the prototypes in a position so that the number of misclassifications is minimized - implying that each prototype should be the closest one to as many samples from its own class as possible.

Schneider et al. extended GLVQ into GMLVQ by introducing an adaptive relevance matrix $\Lambda \in \mathbb {R}^{N \times N}$ in the distance measure [44]. The generalized Euclidean distance can be written as

1 $$\begin{aligned} d^{\Lambda }(\textbf{w}_j, \textbf{x}) = (\textbf{x} - \textbf{w}_j)^\top \Lambda (\textbf{x} - \textbf{w}_j), \end{aligned}$$

where the relevance matrix $\Lambda$ is re-parameterized as $\Lambda = \Omega ^T \Omega$, here with $\Omega \in \mathbb {R}^{N\times N}$, in order to enforce positive semi-definiteness of $\Lambda$ with $d(\textbf{w}_j, \textbf{x}) \ge 0$ for any $\textbf{w}_j$ and $\textbf{x}$ [44]. Additionally, the matrices are normalized such that $\text{ Tr }(\Lambda )$ = $\sum _{i,j=1}^N \Omega _{ij}^2 = 1$. In practice, the auxiliary matrix $\Omega$ is adapted during training. It has the same eigenvectors as $\Lambda$, and the square root of each eigenvalue of $\Lambda$ correspond to the eigenvalues of $\Omega$.

The cost function for GMLVQ is written as a sum over *P* available labeled examples [43]

2 $$\begin{aligned} E \left( \left\{ \textbf{w}_j\right\} _{j=1}^C, \Omega \right)= &   \sum ^{P}_{\mu =1} \phi \left[ \frac{d^{\Lambda }(\textbf{w}_+,\textbf{x}^\mu ) - d^{\Lambda }(\textbf{w}_-,\mathbf {x{^\mu })}}{d^{\Lambda }(\textbf{w}_+,\textbf{x}^\mu ) + d^{\Lambda }(\textbf{w}_-,\mathbf {x{^\mu })}} \right] \nonumber \\  &   \text{ with } \phi (z)=z \text{ in } \text{ this } \text{ work. } \end{aligned}$$

For a given data point $\textbf{x}^\mu$ in the training set, $\textbf{w}_+$ corresponds to the closest prototype of the correct class, and $\textbf{w}_-$ the closest prototype representing a different class. The costs (2) are minimized by placing prototypes close to examples of the same class, and far away from examples of the other classes. At the same time, the optimization with respect to $\Omega$ (and thus $\Lambda$) adapts the weights of the matrix such that directions which discriminate well between the classes are emphasized, and other directions are neglected. In this way, the relevance matrix achieves a mapping into an internal space of the trained GMLVQ model, where the classes are far away from each other, and directions which do not provide as good a separation are collapsed. After training, diagonal and off-diagonal elements of $\Lambda$ quantify the relevance of individual feature dimensions and pairs of features in the classification, respectively.

Conveniently, the directions in feature space which are used to discriminate between the classes are represented by the leading eigenvectors of $\Lambda$, with the corresponding largest eigenvalues. In practice, the training process converges into a low-rank relevance matrix $\Lambda$, where very few eigenvectors dominate the classification [45]. These leading eigenvectors can also be used to visualize the decision space of the trained model by projecting data and prototypes onto the first two or three eigenvectors of $\Lambda$. This projection represents the most discriminative directions of the model. For a three-class problem such as in this work, $\Lambda$ typically converges to two dominating non-zero eigenvalues, and the full decision space can be visualized in two dimensions.

### IRMA

In a specific trained GMLVQ system, the leading eigenvectors of $\Lambda$ with non-zero eigenvalues define a low-dimensional linear subspace of the data space, in which the classification space of that particular solution is realized.

However, the remaining subspace - orthogonal to the one identified by GMLVQ - may still contain class-specific information. The latter subspace is spanned by the set of eigenvectors with eigenvalues $\lambda \approx 0$ of $\Lambda$ in the trained model. This additional class-specific information can be unveiled by retraining the GMLVQ system to distinguish between the same classes, but now restricted to the subspace which was not used for obtaining the initial solution.

This concept is exploited in the recently proposed Iterated Relevance Matrix Analysis (IRMA) [36, 37]. Here, the same classification problem is iteratively retrained and subspace corrected until no class-specific information is left in the data. This retraining is achieved by constructing a correction matrix $\Psi _c$

3 $$\begin{aligned} \Psi _c = \left[ I - \sum _{i=0}^{J-1} \, \textbf{v}^{(i)} \textbf{v}^{(i)\top } \right] \end{aligned}$$

where $\textbf{v}^{(i)}$ are the *J* eigenvectors that have been collected so far and which are projected out in the following training process. In this work, we accumulate a single leading eigenvector per iteration. The correction matrix computed after iteration *i* can then be applied during iteration $i+1$ and achieves a mapping into a lower-dimensional space where the information encoded in the unwanted subspace is disregarded. We implement the correction in this work similarly as in [35], by applying the correction matrix directly on $\Omega$ in each matrix update of the training:

4 $$\begin{aligned} \Omega \rightarrow \Omega \Psi _c. \end{aligned}$$

The stopping criterion for IRMA can be adjusted to the specific needs. In this work, we considered all center-specific signal to be removed when the balanced test accuracy (BAC) in a cross-validation procedure corresponded to random performance. When a level corresponding to random accuracy is reached, the algorithm is not able to meaningfully distinguish between the classes of interest anymore. Eventually, the result of the IRMA procedure is a subspace $V= \textrm{span} \{\textbf{v}_1^{(0)},\textbf{v}_1^{(1)}, \ldots \textbf{v}_1^{(k)}\}$ corresponding to all class-specific information in the data, and a complementary subspace $U = \mathbb {R}^{N} \setminus V$, corresponding to all remaining directions.

As detailed in “Experimental setup”, we applied IRMA on the available HC cohorts, training the system to discriminate between their center of origin. The procedure provided a subspace *V* which contained approximately all center-specific information in the data. Subsequently, a classifier aiming at the actual diagnosis of disorders was trained in the complementary subspace *U*.

### Patients

Four medical centers provided $^{18}$F-FDG PET scans of patients with neurodegenerative brain diseases for this work. These were IRCCS Ospedale Policlinico San Martino (from here on Hospital San Martino, HSM) in Genoa, Tel Aviv Sourasky Medical Center (TAMC) in Tel Aviv, the University Medical Center Groningen (UMCG) in Groningen [46–49], as well as images from a specific camera in the Alzheimer’s Disease Neuroimaging Initiative (ADNI) database. While images in ADNI have been acquired at a wide range of centers, images from each type of camera are acquired with a specified protocol, and are comparable for the purposes of this paper. Data used in the preparation of this article were obtained from the ADNI database (see adni.loni.usc.edu and the Supplementary Material for additional details).

The data from HSM and UMCG was collected based on previous studies or within the framework of clinical practice [46–52]. The data from TAMC corresponds to all patients, controls and relatives included up until January 2024 in a specific study investigating the metabolic brain pattern in DLB patients with and without a GBA mutation, together with their healthy relatives. The local ethical committees approved the aforementioned studies, and informed consent was obtained from all participants or their informed caregivers. Additionally, we included HC and AD subjects from the ADNI database which had been scanned on a GE Discovery STE camera. This scanner was selected due to being relatively modern (FDA approved in 2009) and covering sufficient numbers from each group.

The patients were diagnosed based on current clinical criteria [53–56], and the HCs did not have not have a history of neurological disease. Additional information regarding inclusion criteria and cohort characteristics can be found in the Supplementary Material.

Each center provided roughly age and gender matched healthy controls: $n = 32$ from HSM, $n = 28$ from TAMC, $n = 31$ from the UMCG and $n = 29$ from ADNI ($p = 0.09$ and $p = 0.6$, considering a Kruskall-Wallis and chi-square test, respectively).

Three of the centers each provided one disease cohort for training the disease classification model (training/validation cohort). This comprised $n = 38$ de novo PD patients from HSM, $n = 40$ patients with DLB from TAMC, and $n = 34$ patients with AD from the UMCG. Additionally, the UMCG had $n = 21$ PD and $n = 23$ DLB patients available which were used as a hold out test set together with $n=35$ AD patients from ADNI. The demographics of the patient cohorts are summarized in Table 1.

Furthermore, an additional set of patients ($n = 20$ HC, 20 AD and 20 PD patients) from the UMCG was used only to define the PCA-based feature space. Details of this cohort are listed as the “Ref. group” in Table 1, and corresponds to the space defining reference group in [9].

**Table 1:** Demographic and clinical characteristics of the patient groups, reported as mean (STD), or median [IQR]

	HSM		TAMC		UMCG				ADNI		Ref. group		
	HC	PD	HC	DLB	HC	AD	PD	DLB	HC	AD	HC	AD	PD
n	32	38	28	40	31	34	21	23	29	35	20	20	20
Sex, male %	66	61	54	90	61	62	67	78	46	71	75	60	75
Age, years	66.5 (6.9)	72.2 (6.5)	64.4 (13.9)	71.2 (6.8)	65.2 (4.5)	65.4 (7.9)	66.4 (11.0)	72.0 (7.3)	68.5 (4.2)	75.8 (6.8)	65.3 (6.9)	68.3 (8.2)	64.4 (7.4)
Disease duration$^{1}$	−	1.0 [0.8, 2.0]	−	3.0 [1.4, 5.0]	−	2.0 [1.0, 3.0]	4.0 [1.8, 7.3]	2.0 [1.0, 3.0]	−	3.1 [0.8, 4.3]	−	2.9 (2.4)	4.7 (3.5)
MMSE$^{2}$	−	28.1 (2.1)	−	24.9 (4.6)	−	−	−	−	29.9 (0.3)	21.4 (5.2)	29 (0.8)	23.8 (3.8)	28.3 (1.2)

$^{1}$Time in years since disease onset

$^{2}$Mini-Mental State Examination score

**Table 2:** Summary of image acquisition protocol per center

	HSM	TAMC	UMCG	ADNI
Scanner	Biograph Truepoint 16	Discovery MI	Biograph mCT40	Discovery STE
			Biograph mCT64	
Manufacturer	Siemens	GE Healthcare	Siemens	GE Healthcare
Dose $^{18}$F-FDG (MBq)	185-200	370	200	167-204
Uptake time (min)	45	45-60	30	30
Acquisition time (min)	10	12	5	30 ($6 \times 5$)
Scanner generation$^{3}$	2009	2015	2012	2009
Reconstruction algorithm	OSEM	VPFXS	OSEM3D+ToF+PSF	3D Iter
Iterations, subsets	6i16s	6i17s	3i21s	4i20s
Voxel size	$1.33 \times 1.33 \times 2$	$1.17 \times 1.17 \times 2.79$	$2 \times 3.18 \times 3.18$	$2.00 \times 2.00 \times 3.27$
Gaussian smoothing (mm)	8 (post-processing)	8 (post-processing)	8 (on-camera)	8 (post-processing)

$^{3}$Year of FDA approval

### $^{18}$F-FDG PET imaging and feature extraction

Each center acquired and reconstructed the images according to the local hardware and protocol. Main parameters are summarized in Table 2.

The UMCG patients were acquired on a Siemens Biograph mCT40 or mCT64 PET/CT camera, with 200MBq $^{18}$F-FDG, 30min uptake time, and 5min acquisition time. The images were reconstructed with the OSEM3D algorithm (3 iterations, 21 subsets), time-of-flight, point-spread-function, and a matrix size of 256 (corresponding to a voxel size of $2 \times 3.18 \times 3.18$ mm). The images were intrinsically smoothed with an 8mm full width at half-maximum Gaussian filter.

The HSM patients were acquired on a SIEMENS Biograph 16 PET/CT with a total axial field of view of 15 cm and no interplane gap space. A dose of 185-200MBq was used in combination with 45min uptake time and 10min acquisition time. Images were reconstructed using the OSEM algorithm (6 iterations, 16 subsets) with a reconstructed voxel size of $1.33 \times 1.33 \times 2.00$ mm$^3$.

The TAMC patients were acquired on a GE Discovery MI after an injection of 370MBq on average and 45-60 min uptake time. The images were reconstructed with the VPFXS algorithm (6 iterations, 17 subsets) and a reconstructed voxel size of $1.17 \times 1.17 \times 2.79$ mm$^3$.

The ADNI subjects were imaged with 167-204MBq on a GE Discovery STE camera with an aquisition time of 30 minutes reconstructed into six frames. The images were reconstructed with a 3D iterative reconstruction algorithm using 4 iterations and 20 subsets, with a voxel size of $2.00 \times 2.00 \times 3.27$. We considered the averaged, co-registered images as provided by ADNI.

The scans from all centers were spatially normalized to an $^{18}$F-FDG PET template in Montreal Neurological Institute (MNI) brain space using the SPM12 software (Wellcome Centre for Human Neuroimaging, London, UK) implemented in MATLAB (version R2019a; MathWorks, Natick, MA, USA). The scans from HSM, TAMC and ADNI were smoothed with an 8mm full width at half-maximum Gaussian filter at this step, to reach the same level of smoothing as the UMCG images.

Each scan was masked to include grey matter areas only, where the mask was obtained by considering the 116 regions included in the AAL atlas [57] as implemented by the WFU PickAtlas Tool. Thereafter the $^{18}$F-FDG PET scans were intensity-normalized using global mean normalization [18]. This involves normalizing the average voxel value into unity for each subject, by dividing each voxel value by the mean uptake of that subject. All scans were flattened into vectors for further use.

A PCA-based coordinate system was defined by applying PCA on the set of vectors corresponding to the preprocessed space defining reference cohort. Feature vectors for all data were then obtained by projecting the preprocessed image vectors onto the given principal components, including those which covered 80% of the variance in the space defining reference group. This eventually resulted in vectors of 31 features for each preprocessed patient scan. For the actual model training, features were z-scored based on the parameters of the HCs of the space defining reference group, to obtain roughly comparable magnitudes.

### Experimental setup

We determined the subspace representing the center difference by training IRMA to classify the center label of the HC subjects of the four medical centers. Hereafter, we denote this subspace as *V*. We removed one eigenvector per iteration, obtained by training using all available HC, and applied 10 times repeated 10-fold cross-validation after each IRMA iteration to estimate how much center-specific information was left. We used the balanced accuracy score (BAC) as our primary evaluation metric, equivalent to the average sensitivity (recall) of each class, with random guessing corresponding to $BAC = 1/{n_{classes}}$ [58]. We recorded how many features were significantly different $(p<0.05)$ in the HC cohorts before and after harmonization, considering a Kruskal-Wallis test with and without Bonferroni correction. For this purpose, we applied the correction matrix directly on the feature vectors.

We then addressed a disease classification problem, where each disease cohort (PD, DLB and AD) was acquired at one of three centers. For this purpose, we trained a set of GMLVQ models - initially unrestricted, and thereafter increasingly corrected for the center-specific information, where the *i*-th model was corrected using *i* accumulated eigenvectors from the HC classification problem. We cross-validated each *i*-th model to investigate how the separation of the disease classes was influenced by increasing correction.

We measured the contribution of center-specific information in the disease models by considering the subspace angle as defined in [59]. This measures the largest possible alignment between any two vectors from the two subspaces (one per subspace) as the first principal angle, and the following angle is the largest possible alignment between any two vectors orthogonal to the first ones considered. We considered the angle between *V* and the subspace formed by the two leading eigenvectors of the *i*-th trained disease model, $D_i= \textrm{span} \{\textbf{v}_i^{(0)},\textbf{v}_i^{(1)} \}$. We obtained $D_i$ by training the *i*-th disease model once using all data. This resulted in two unique angles, considering that $D_i$ is two-dimensional. Angles of 90 degrees would indicate that no center-specific information was used in the disease solution, and 0 degrees would indicate a complete overlap of information. In other words, the subspace angle indicates how much bias originating from center-specific signal was present in the trained disease model.

We evaluated the impact on cross-center classification performance by using a test set, where each disease class in the test set was scanned at a different center than the same disease class in the training set. We reported how a GMLVQ model trained with all available training data performed on the test set with and without harmonization. In addition to IRMA-based harmonization, we also evaluated test scores obtained with two alternative harmonization methods: center-wise z-scoring and ComBat. Here, center-wise z-scoring entails z-scoring the features separately for each center, where the mean and standard deviation of each feature was estimated based on that center’s HC cohort. The harmonization parameters for ComBat were obtained similarly using the Python NeuroCombat implementation with default parameters [27].

**Fig. 1:**
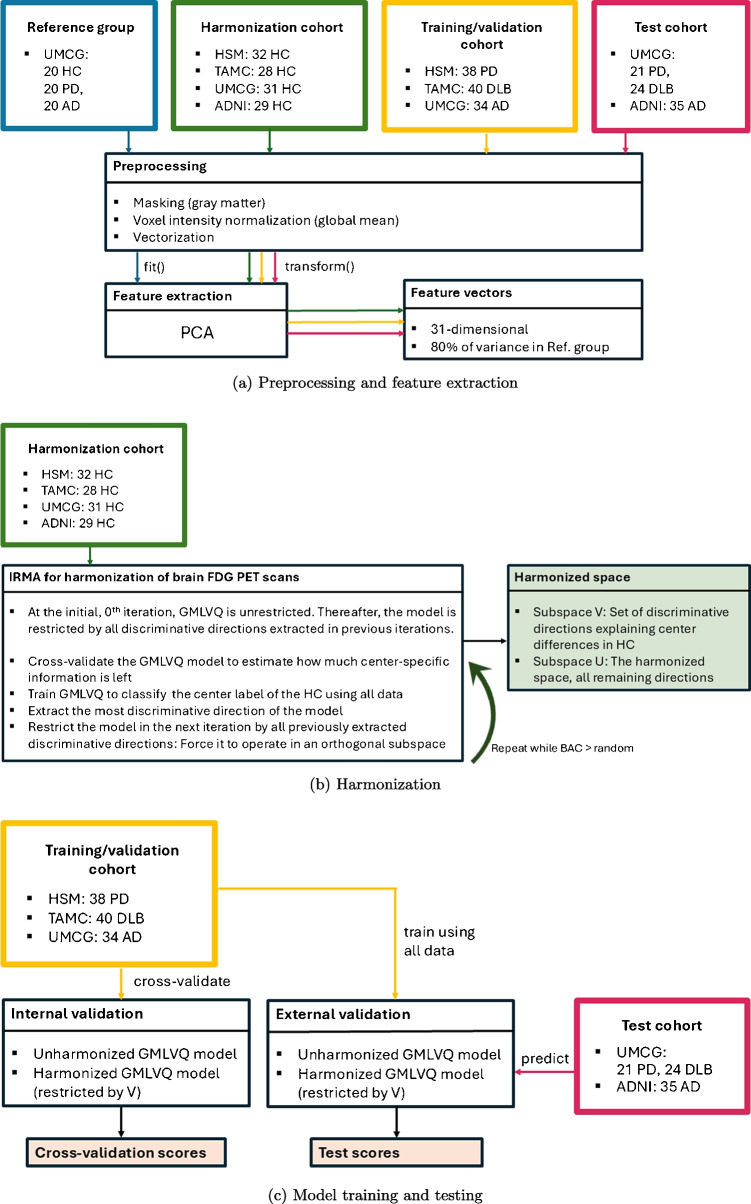
Overview of the computational pipeline

An overview of the computational pipeline can be seen in Fig. 1 with preprocessing and feature extraction in Fig. 1a, harmonization step in Fig. 1b, and model training/validation and testing in Fig. 1c.

Next, we investigated whether the center correction removed disease-specific information. Removing too much information would, of course, deteriorate the quality of the data and its signal. For this purpose, we trained an uncorrected GMLVQ model to classify data in a *single center* setting, and compared the cross-validated results with those of a GMLVQ model corrected by *V*. We specifically used single-center settings to address this question, as we are sure that between-center bias would not create an inflated performance in an uncorrected system. However, if disease-related information has been removed, we would see a deterioration in performance in the corrected system. To perform these experiments, we used the full set of patients available at each center. For the UMCG this included HC, AD, DLB, and PD (listed in Table 1), for HSM, we compared HC vs PD, for TAMC we considered HC vs DLB and for ADNI HC vs AD. Additionally, we measured the subspace angle between *V* and the nonzero eigenvectors obtained by training a model to classify AD, DLB and PD using data from the UMCG only.

**Fig. 2:**
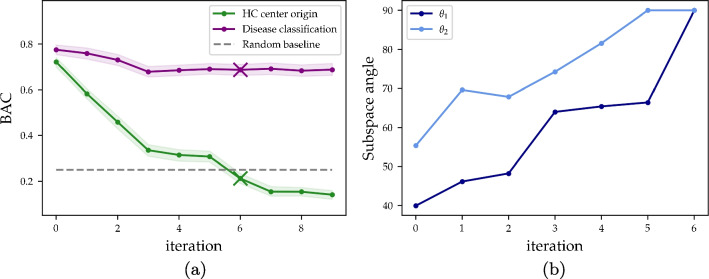
(a) Balanced accuracy (BAC) per iteration of IRMA for classifying the center origin of HC subjects of the four centers (green curve), as well as disease classification problem (purple curve), with $95\%$ confidence intervals within shaded regions. For each iteration, the system was increasingly corrected for the center-specific information present in the data. (b) Subspace angles in degrees between the model space of a trained disease model, increasingly corrected, and the six-dimensional subspace *V* found to represent the center difference

Finally, we visualised our results in voxel space. Any data point, including the prototypes of each class, can be visualised similarly as in previous works applying GMLVQ on $^{18}$F-FDG PET data [9]. If we denote the eigenvectors resulting from the PCA procedure as *G*, and a preprocessed image as *X*, we can approximately project a feature vector $\textbf{x}$ back into voxel space by $X \approx \textbf{x} G^T$, assuming the feature vectors were obtained as $\textbf{x} = XG$. We can also visualize corrected feature vectors in voxel space, or a representation of corrected images, $X_{corr}$, according to

5 $$\begin{aligned} X_{corr}= &   \textbf{x} \Psi G^T = X G ( I- \sum _{j=0}^{J-1} \textbf{v}^{(j)} \textbf{v}^{(j)T}) G^T\nonumber \\= &   X - x \sum _{j=0}^{J-1} \textbf{v}^{(j)} \textbf{v}^{(j) T} G^T \end{aligned}$$

Considering the last expression of this equation, we see that the center-corrected scan can be expressed as the original scan *X* (preprocessed, and disregarding the information loss occurring from projecting the data point into and back from PCA space) minus the information in *X* which resides in *V*. Note that the eigenvectors obtained from the PCA procedure are orthonormal, resulting in $G G^T = I$. In our results, we visualized the mean feature vector from each group, showing the corresponding original scan *X*, the removed profile resulting from the correction procedure $X_{rem}$ = $\textbf{x} \sum _{j=0}^{J-1} \textbf{v}^{(j)} \textbf{v}^{(j)T} G^T$, and the corrected version $X_{corr}$. For these visualizations, we undid the scaling of the feature vectors and applied a voxel-wise z-scoring based on the values of the HCs in the space defining reference group.

All GMLVQ and IRMA systems were trained using 30 iterations of waypoint gradient descent, with identity activation function and initial step sizes of 1 and 2 for the prototypes and relevance matrix, respectively. All other parameters were kept at their default values as implemented by the Python sklvq library [60] v0.1.2.

## Results

Cross-validated balanced accuracy (BAC) versus the iteration of IRMA can be seen in the left panel of Fig. 2, where iteration 0 denotes an unrestricted system. Initially, GMLVQ was able to classify the HCs of the four centers highly accurately with a BAC of 0.72 (AUC = 0.89). It required six iterations of IRMA, corresponding to a six-dimensional subspace *V*, until the center classification (green) reached random accuracy (BAC $\le 1/4$). Class-specific recall curves can be viewed in Fig. 1 in the Supplementary Material.

**Fig. 3:**
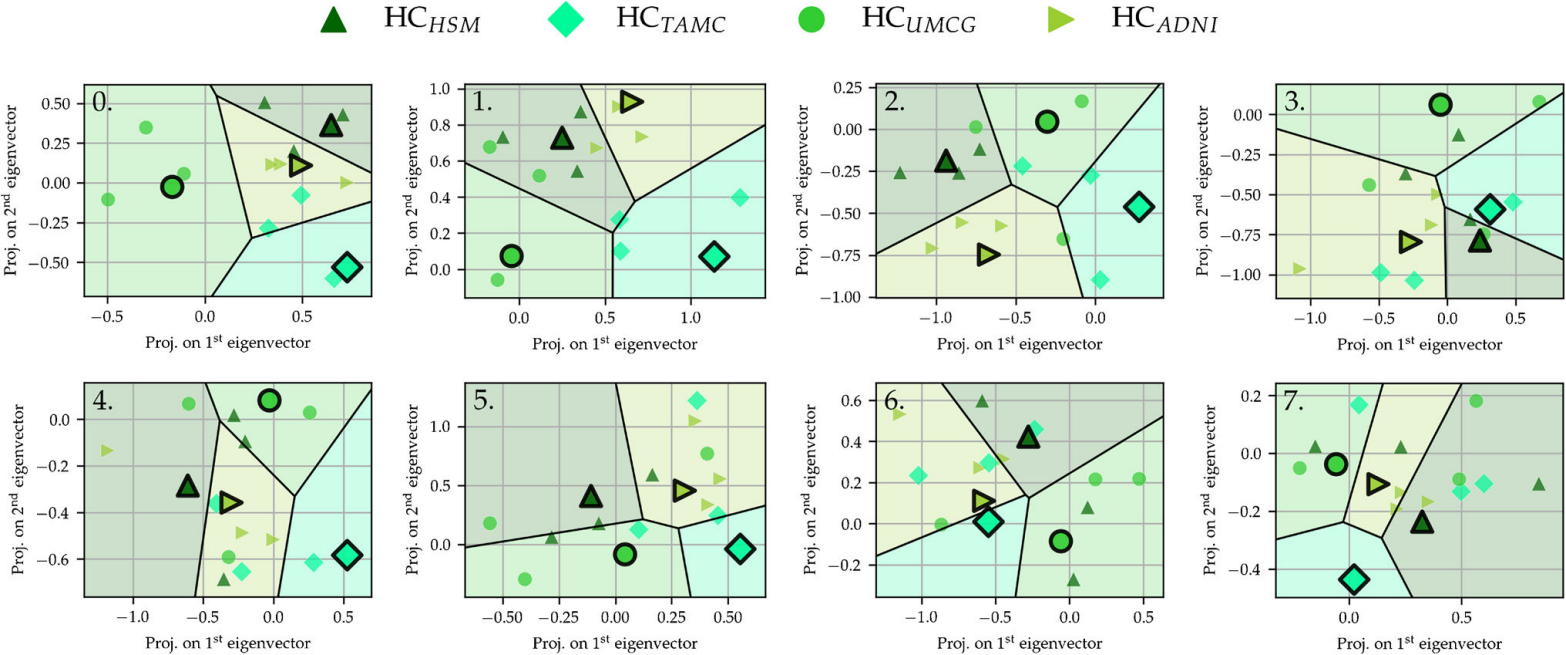
IRMA applied to center classification in healthy controls: Discriminative visualizations of an arbitrarily chosen test set of the cross-validation procedure, projected onto the two leading eigenvectors of $\Lambda$ after training. The system was trained to classify the center origin of the HC data, shown for the first seven iterations of IRMA, where the system was increasingly corrected for the center-specific information

Figure 3 shows one arbitrarily selected test set from the cross-validation of the IRMA procedure. The model was trained to discriminate between the center label of the HCs, whereafter the test set was projected onto the two leading eigenvectors of the relevance matrix of the trained model. It can be seen that the distributions were initially well separated, but move closer and closer to each other, until they overlap and were quite indistinguishable in iteration six. The obtained separation between the classes reflects the cross-validated BAC curve in Fig. 2. Hereafter, we assumed that the subspace *V* representing the center-specific information corresponded to the first six accumulated eigenvectors from the IRMA procedure. This point, where we have performed a sufficient number of iterations for full harmonization, is highlighted with a cross in Fig. 2. Figure 2 in the Supplementary Material displays discriminative visualizations of applying IRMA on the full HC data set.

The purple curve in Fig. 2a shows the BAC of classifying the three disease classes, where iteration *i* denotes a model which was corrected using the *i* first accumulated eigenvectors of the HC classification problem. An uncorrected system achieved a cross-validated BAC of 0.78 (AUC = 0.94), with BAC = 0.69 (AUC = 0.84) at iteration six, whereafter the performance remained stable for further iterations.

The subspace angles between $D_i$ - the model space of the *i*:th trained disease model - and the center subspace *V*, can be seen in Fig. 2b. An uncorrected disease model obtained angles of 40 and 55 degrees, respectively, meaning that roughly half of the information it used to discriminate between the diseases came from *V*. The contribution of *V* in the trained disease model decreased the more the training process was restricted, with the sixth model being fully restricted by *V*.

Before correction, 19 features were significantly different ($p<0.05$) between the four HC cohorts. After correction, six significantly different features remained. If we considered the same significance level with Bonferroni correction, there were 9 features different between the centers before correction, and none after. It can be noted that among the first 10 features, corresponding to the first 10 principal components (together explaining $51\%$ of the variance of the space defining reference group), feature 0, 1, 2 and 7 displayed $p \le 0.001$ in the unharmonized data. Hence, center differences were not only present in principal components explaining small amounts of variance, but also in the most dominant ones. Figure 3 in the Supplementary material shows histograms of the distributions before and after correction for the first three features.

**Fig. 4:**
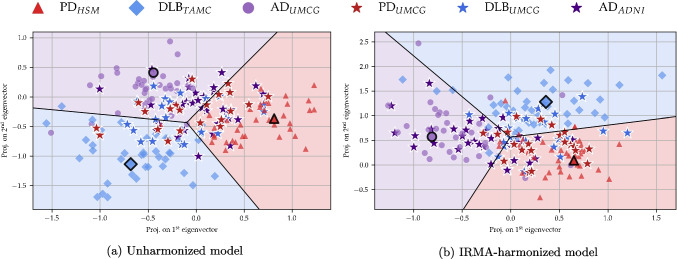
Discriminative projection of a GMLVQ model trained to distinguish between PD from HSM, DLB from TAMC, and AD from the UMCG. Unseen PD and DLB patients from the UMCG were projected in as a test set, marked as red and blue stars. The prototype for each class is marked with a black border. (a) The model was trained without any center-harmonization (BAC = 0.41 for the test cohort). (b): IRMA-harmonized model (BAC = 0.59)

**Table 3:** Performance scores on unseen test cohorts for the disease classification model - uncorrected, or center-harmonized using three different methods: IRMA, ComBat or z-scoring

	PD		DLB		AD			
	Precision	Sensitivity	Precision	Sensitivity	Precision	Sensitivity	BAC	AUC
No harmonization	0.32	0.48	0.38	0.26	0.53	0.49	0.41	0.55
IRMA	**0.42**	**0.71**	**0.62**	0.43	0.81	**0.63**	**0.59**	**0.79**
ComBat	0.38	0.67	0.60	**0.52**	**0.82**	0.51	0.57	0.73
z-score	**0.42**	0.67	0.43	0.26	0.69	**0.63**	0.52	0.70

Bold font (highest performance)

Next, we trained our disease models using all available training data, and evaluated cross-center classification performance using a test set where each group was scanned at a different center than the same disease in the training set. Discriminative projections can be seen in Fig. 4. Here, the training data is displayed as red triangles (PD from HSM), blue diamonds (DLB from TAMC) and purple circles (AD from the UMCG). The test data was projected in as red (PD from the UMCG), blue (DLB from the UMCG) or purple (AD from ADNI) stars. In Fig. 4a, reflecting the decision space of a model trained without any center correction, we see that the test data scatters generously into each of the three sections. This was also reflected in a BAC of 0.41 (AUC 0.55), close to random guessing. The decision landscape of a model center-corrected by IRMA can be seen in 4b. Here, both test groups originating from the UMCG moved away from AD space - towards their own corresponding prototype, and with a decreased tendency to prioritize information from its own scanner. The number of PD and DLB test samples predicted to be AD decreased from 29 and 39% to 14 and 9%, respectively. The sensitivity (recall) for the three classes increased from 0.48, 0.26 and 0.49 to 0.71, 0.43 and 0.63, resulting in an overall BAC of 0.59 (AUC $$ = 0.79$$). A summary of the performance scores can be seen in Table 3. The IRMA-corrected system performed significantly better than a model trained with no harmonization for all measures. The table also includes scores obtained by two alternative harmonization methods: ComBat- and z-score-based harmonization. Center-wise z-score harmonization obtained a BAC of 0.52 (AUC = 0.70). IRMA-harmonization displayed an advantage over ComBat-harmonization, with BACs of 0.59 vs 0.57 and AUC = 0.79 vs 0.73. The IRMA-corrected model classified PD and AD patients slightly better than the ComBat-corrected model (sensitivity 0.71 vs 0.67 and 0.63 vs 0.51, respectively), while the latter was slightly better at classifying DLB patients (sensitivity 0.43 vs 0.52).

Performance scores stayed approximately constant when training a GMLVQ classifier in a single center setting, with and without correction by *V*. For the UMCG (HC, AD, PD, DLB) and HSM (HC vs PD) the BAC stayed identical at 0.66 and 0.75, respectively. For TAMC the scores were 0.97 and 0.96 without and with restriction by *V* (HC vs DLB). For ADNI, the performance decreased from BAC = 0.91 to BAC = 0.86, indicating a slight loss of information for this data. The largely preserved classification accuracy in a single-center setting indicates that the harmonization procedure did not remove any critical information. Measuring the subspace angle between *V* and the model space obtained when training a GMLVQ model to distinguish between AD, PD and DLB from the UMCG only, resulted in angles of 60 and 70 degrees, respectively. This indicates that the overlap between the center-specific information and the information which would be used to distinguish between the diseases in a single-center setting is quite limited.

**Fig. 5:**
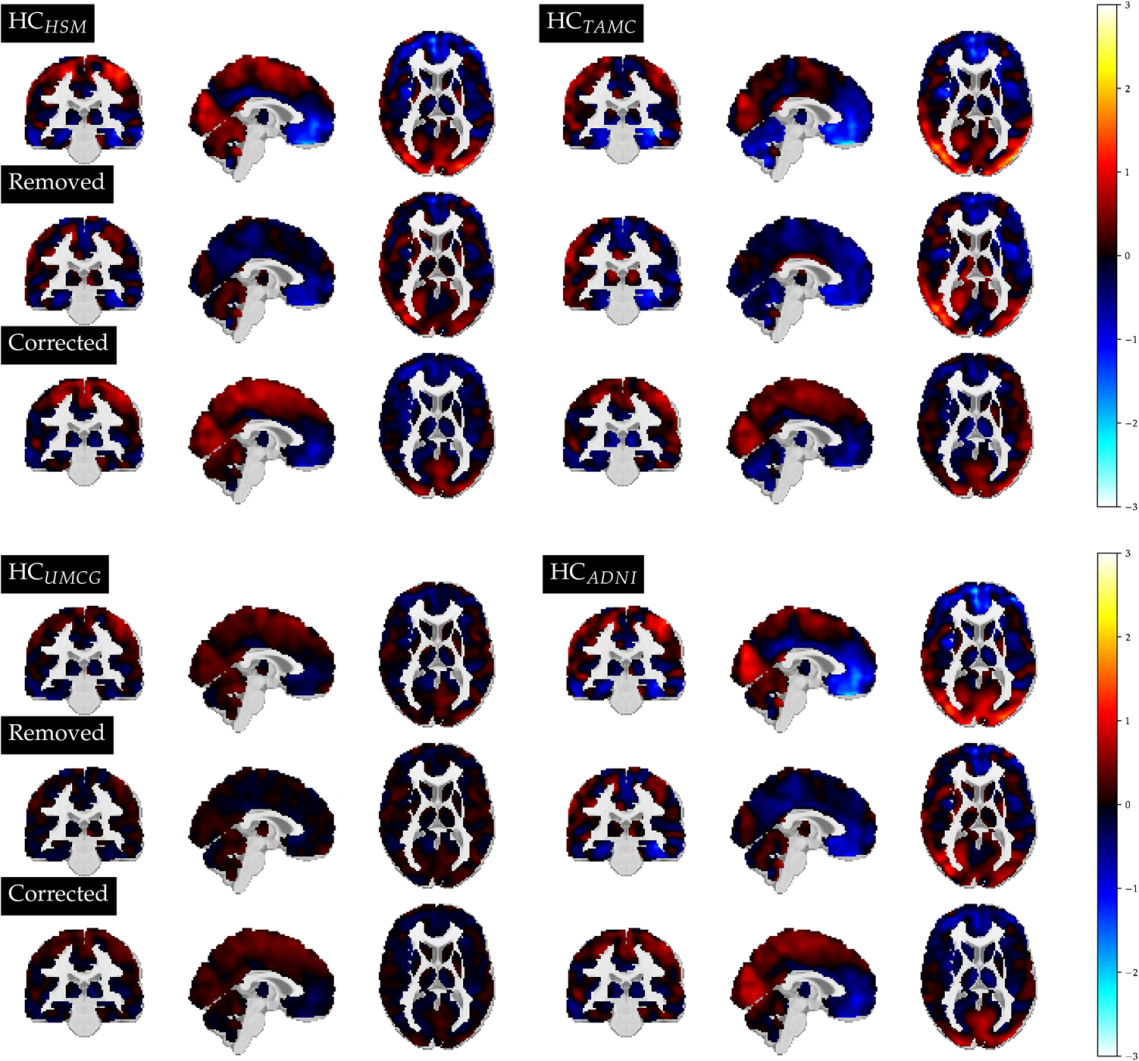
Voxel-space visualization of the average HC per center. For each center, the original scan is shown on each top row, the profile which was removed in the correction in the middle row, and the corresponding corrected scan at the bottom row. The voxel values are displayed as a z-score with respect to the controls of the space defining reference group

**Fig. 6:**
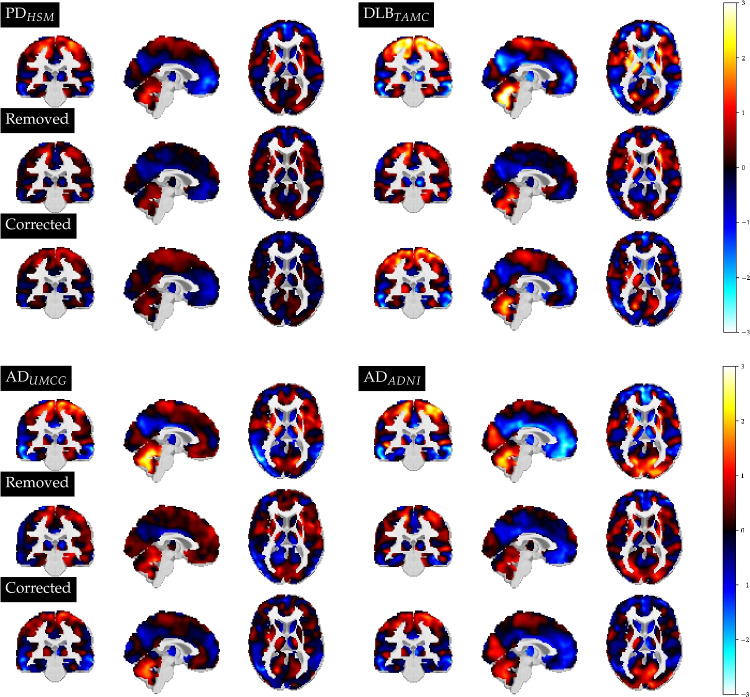
Voxel-space visualization of an average disease sample from each center. For each group, the original scan is shown on the top row, the profile which was removed in the correction in the middle row, and the corresponding corrected scan at the bottom row. The voxel values are displayed as a z-score with respect to the controls of the space defining reference group

Figure 5 displays voxel-space visualizations of our HC subjects before and after correction, as well as the profile which was removed for that specific image. The figure displays the reconstructed average feature vector, and voxel values are represented as a z-score based on the HCs of the space defining reference group. In each top row we see slices of the original image, and each bottom row represents the center-harmonized version, after applying a correction by *V*. The profile in the middle row represents the contribution of *V* in the original scan, according to Eq. 5. As an example, it can be seen that the metabolism in the frontal part in the HCs from HSM, TAMC and ADNI seemed notably lower than in HCs from the UMCG, while the uptake in the Cerebellum of TAMC was lower than for the other three centers. In the corrected scans, the profiles looked more similar, and the low frontal metabolism in subjects from HSM, TAMC and ADNI was relatively increased.

Figure 6 displays the same voxel space profiles, but for the average disease subject from each center. Low values represent relative hypometabolism (blue/light blue), and high values relative hypermetabolism (red/yellow). Here, the removed profile again represents the part of the information residing in *V* for that patient, but this time it did not lead to highly similar corrected scans. Hence, the metabolic expression which is characteristic for each disease was preserved. It can also be noted that the AD patients from the UMCG and ADNI, which uncorrected had visible differences e.g. in the frontal part of the brain, are more comparable in the corrected profile.

## Discussion and conclusion

Center-specific differences in brain $^{18}$F-FDG PET scans are prominent, as demonstrated by a near-perfect classification accuracy when determining the center origin of healthy controls. We demonstrated how these differences - if uncorrected - introduce bias in machine learning models trained on a clinically relevant problem. Using the basic assumption that age and sex-matched HC cohorts should have similar distributions, we showed how IRMA can be used to learn and disregard center-specific signal in a multi-center setting. No meaningful center-specific information was left in the feature vectors after the harmonization, evidenced by the random performance when discriminating between the HC cohorts in a cross-validation procedure. The harmonization substantially reduced the number of features which were significantly different in the HC cohorts according to a Kruskal-Wallis test. This was 19 before and 6 after harmonization considering $\alpha = 0.05$, or 9 before and none after considering the same significance level with Bonferroni correction. IRMA identified a 6-dimensional subspace representing center differences for the four centers considered. This subspace is denoted as *V*, where the information in *V* is projected out and removed from consideration in the harmonization procedure. Thereafter, any subsequently trained models (or the data itself) can be restricted to the complementary subspace *U*.

After training a model to classify PD (from HSM), DLB (from TAMC) and AD (from the UMCG), we used unseen PD and DLB patients from the UMCG together with AD from ADNI as a test set. The harmonization significantly improved cross-center classification performance, as illustrated by an increased BAC of 0.41 vs 0.59, and AUC increasing from 0.55 to 0.79 compared to no harmonization (see Table 3). This was in stark contrast to the scores obtained for internal validation, where the cross-validation score of the uncorrected model (BAC = 0.78, iteration 0) clearly did not represent a realistic estimate. The center-bias of the unharmonized model was formally quantified by the concept of subspace angles, where we observed a large overlap between the information used by the unharmonized disease model and *V*. While the IRMA-harmonized test score may still seem low, the performance is in line with the class-specific recall obtained for these diseases in a previous work including the same diseases in a single-center setting [9]. Furthermore, PD and DLB are part of the same disease spectrum (Lewy Body Diseases) and share metabolic features, representing a complex classification problem [61]. Therefore, these results are in line with current literature.

The aim of our study was to demonstrate the properties of IRMA and the preservation of disease-specific information while correcting for center differences. It was not our goal to validate biomarkers for diagnostic or prognostic purposes. For such a purpose, larger cohorts of $^{18}$F-FDG PET scans would be necessary, with more extensive clinical information. The classification accuracy of our model may have been influenced by clinical differences between cohorts, such as disease duration or severity (i.e. PD patients from HSM were de novo, with a median duration of motor symptoms of 1 year, compared to a disease duration of 4 years in the UMCG cohort).

This work showed a clear advantage of IRMA-based harmonization compared to center-wise z-scoring, where the model was not able to classify DLB patients reliably (sensitivity 0.26). For our setting, IRMA-based harmonization was also slightly better than ComBat (BAC 0.59 vs 0.57, AUC 0.79 vs 0.73). It should be noted, however, that this experiment was an illustrative application of the methods rather than a formal comparison, and performance scores may vary between cohorts and experimental setups.

To compare IRMA to statistics-based approaches from a conceptual point of view, the basic idea behind the latter is to apply a shift and scaling per feature to align the distributions. These statistics-based methods likely have an advantage when the number of features is relatively small, so that the data space is populated enough in order to estimate the parameters robustly. These methods then harmonize the distributions without eliminating any feature or directions in feature space. They also preserve the internal order between data points for a single center, while they may change the order of comparable data points between centers, as each center is shifted and scaled separately. Furthermore, a data space with a fixed number of points becomes increasingly sparse with higher dimensionality, and the number of samples needed to estimate a multivariate distribution grows exponentially [33]. In the intersection of molecular neuroimaging and machine learning, it may be especially challenging to obtain sufficient (HC) sample sizes for high-confidence estimation of harmonization parameters. It is indeed in the high-dimensional domain that IRMA should provide a critical advantage. The algorithm learns a set of discriminative directions, which meaningfully provide a separation between HC cohorts of the different centers. The resulting subspace *V*, and corresponding correction matrix $\Psi$, is then *the same* for all centers and data considered. Hence, in principle the IRMA harmonization procedure does not change the data, but merely points to one subspace where the information *is* comparable between the centers, and another subspace where the signal *is not* comparable. In this way, IRMA mitigates the main pitfall with statistics-based methods, where in the worst case scenario you introduce new bias in the data after harmonization in case the parameters are not well estimated (i.e., shifting or scaling some features too much or too little). In addition, IRMA does not pose requirements on the properties of the features, such as Gaussianity or statistical independence. As a disadvantage, IRMA is not suitable for problems where the signal of clinical interest (e.g., discriminating between diseases) is correlated with the center-specific signal. It is also possible that IRMA would not detect all center signal in case of too small a sample size, in which case only part of the center bias could be removed. Additionally, the IRMA pipeline needs to be refitted for each new center added.

An inherent question arises with this novel way of harmonizing data: What if *V* contains critically important information, as we are not allowed to consider this subspace after the harmonization? Our experiments involving classification in a single-center setting showed that in general no essential disease specific information was removed by the harmonization process, as indicated by a mostly constant performance with and without correction by *V*. There was some overlap of information between *V* and information used by a GMLVQ model to discriminate between AD, PD and DLB using data from a single center (UMCG), with subspace angles of 60 and 70 degrees, respectively. This indicates that the information used to distinguish the three diseases was to a small extent related to information residing in the center difference subspace *V*, and had a minor negative impact the ADNI data (HC vs AD). However, the single center experiments with the other three centers were not affected negatively, supporting the notion that IRMA generally preserved the disease-specific information.

Furthermore, IRMA provides full control over the harmonization procedure. This is for one part due to the linear properties of GMLVQ and IRMA, providing analytical expressions for the harmonization procedure. This can be considered in opposition to black-box deep learning methods, such as the generative adversarial network recently proposed for harmonization by Haberl et al. [62], which however is capable of harmonizing at the image-level. Second, data from all centers, before and after correction, resides in a fixed data space. These properties enable direct comparison between subspaces, e.g. measuring the amount of unwanted information in an uncorrected model, as well as direct quantification of the information which is removed from each scan.

Some limitations of this work need to be addressed. Naturally, the IRMA harmonization procedure may also suffer from the same data availability limitations as statistics-based methods, despite possibly being more robust. In case the center difference subspace is not well estimated, training a model in the complementary subspace may lead to the model focusing on suboptimal information. Moreover, the procedure does remove a subspace of the data space from consideration. In our work, the remaining 25-dimensional subspace was more than sufficient to maintain essential information, but there may be situations in lower-dimensional settings where the method would remove too large a portion of the original data space. Furthermore, a large portion of center-specific effects could probably be reduced in case efforts toward prospective image-level harmonization would be taken, e.g. based on EARL guidelines. Therefore, in the optimal scenario, EARL-based harmonization could be conducted prospectively, with IRMA-based harmonization additionally applied on features derived retrospectively from the images. It should also be noted that IRMA harmonizes feature vectors derived from PET images, but not the images themselves, which does not allow for visual assessment of harmonized (raw) images.

Furthermore, while we age and gender matched our HC cohorts, it is possible that the HC cohorts still contained other bias than only the center-specific signal. Similarly, it is not excluded that our disease model contained a small amount of bias relating to sex, age, ethnicity or other differences. In this work we did not look further into these nuances.

Another limitation of the method is the dependence on HC cohorts in itself, which are costly to acquire, and requires additional permission from medical ethical committees. Future work should estimate the number of HCs the method needs at a minimum in order to identify the center difference subspace robustly. Additionally, forthcoming studies should investigate whether *V* could possibly be estimated using the Hoffman 3D brain phantom, which - if affirmative - would remove the necessity for HC cohorts. Another option may be computationally simulated and reconstructed healthy control scans. Future studies may also validate the application of the method for other types of feature vectors or other tracers. It would also be interesting to apply the methodology to databases where steps have already been taken to minimize center differences, such as the full set of scans in ADNI.

In conclusion, IRMA-based center harmonization enables merging of high-dimensional data sets in molecular brain imaging, taking steps towards further progress in the largely unexplored area of machine learning in clinical nuclear medicine.

## Supplementary Information

Below is the link to the electronic supplementary material.

Supplementary file 1 (pdf 1645 KB)

## Data Availability

Software for our method is freely available at https://github.com/SofieLovdal/IRMA-harmonization.
