# Supplementary material for: IRMA: Machine learning-based harmonization of 18F-FDG PET brain scans in multi-center studies
Source: Eur J Nucl Med Mol Imaging. 2025 Feb 18;52(8):2941–58. doi: 10.1007/s00259-025-07114-4 (PMC12162725; doi:10.1007/s00259-025-07114-4)
Supplement: Supplementary file 1 — (pdf 1645 KB) [file 259_2025_7114_MOESM1_ESM.pdf]

Supplementary Material

IRMA: Machine learning-based harmonization of ^{18}F-FDG PET brain scans in multi-center studies.

Additional details regarding experimental setup

NeuroCombat version 0.2.12 was used to implement the Combat-related experiments, using default parameters (Empirical Bayes, parametric adjustments, and average as reference batch).

Additional details regarding imaging and patient cohorts

Part of the data used in the preparation of this article were obtained from the Alzheimer's Disease Neuroimaging Initiative (ADNI) database (adni.loni.usc.edu). The ADNI was launched in 2003 as a public-private partnership, led by Principal Investigator Michael W. Weiner, MD. The original goal of ADNI was to test whether serial magnetic resonance imaging (MRI), positron emission tomography (PET), other biological markers, and clinical and neuropsychological assessment can be combined to measure the progression of mild cognitive impairment (MCI) and early Alzheimer's disease (AD). The current goals include validating biomarkers for clinical trials, improving the generalizability of ADNI data by increasing diversity in the participant cohort, and to provide data concerning the diagnosis and progression of Alzheimer's disease to the scientific community. For up-to-date information, see adni.loni.usc.edu.

From ADNI, we included subjects from a camera which was reasonably recent and also had a sufficent number of subjects available: The GE Discovery STE. ADNI has a specified PET acquisition protocol, so even though the images may come from different centers, they were all acquired and reconstructed with the same protocol. Many subjects in ADNI have been imaged several times during the follow-up. We included the first scan only of each subject, and considered the averaged and co-registered version of the six frames as provided by ADNI.

The data from HSM was collected within the framework of clinical practice from 2014 onwards [1], or in connection to specific studies [2], and this cohort corresponds directly to the one used previously in [3]. The data from TAMC corresponds to all patients, controls and relatives included up until January 2024 in a specific study investigating the metabolic brain pattern in DLB patients with and without a GBA mutation, together with their healthy relatives. The data from the UMCG corresponds to a set of previous studies [4–7] as well as additional patients collected retrospectively within the framework of clinical practice. The UMCG cohort in this work directly corresponds to the cohort used to classify ^{18}F-FDG PET scans in a single center setting in [8].

The HC groups from the four centers were roughly age-matched by excluding the few oldest controls from HSM and ADNI and the few youngest controls from the UMCG and TAMC. For the latter cohort, healthy relatives of DLB subjects (excluding the ones carrying a GBA mutation) were merged and considered HCs. The HC from UMCG, HSM and TAMC did not have a history of neurological or psychiatric disease or other chronic illnesses and were not taking psychoactive medication. The HC from ADNI subjects were included as the ADNI Cognitively Normal (CN) subjects. The specific inclusion criteria are documented via ADNI and include a MMSE of at least 24, age between 55-90, and no significant neurological disease [9]. Overall, we consider the HC cohorts to reflect the general elderly population.

The disease cohorts from UMCG, HSM and TAMC were diagnosed according to current clinical criteria.

A separate set of patients was used for the space defining reference group, as applying PCA directly on all available data would introduce potential overfitting effects at the feature extraction step.

The subjects acquired at the UMCG were scanned on two different mCT cameras. These Siemens mCT40 and mCT64 systems are nearly identical, with the same PET component, but differing number of CT slices (40 vs 64). Since CT has a significantly higher resolution than PET, the resulting differences in attenuation correction would be negligible, and we therefore assumed that images from these two cameras at the UMCG were directly comparable.

Additional results

References

[1] Orso, B., Arnaldi, D., Girtler, N., Brugnolo, A., Doglione, E., Mattioli, P., Biassoni, E., Fancellu, R., Massa, F., Bauckneht, M., *et al.*: Dopaminergic and serotonergic degeneration and cortical [18F] fluorodeoxyglucose positron emission tomography in de novo Parkinson's disease. Movement Disorders **36**(10), 2293–2302 (2021)

[2] Mattioli, P., Pardini, M., Famà, F., Girtler, N., Brugnolo, A., Orso, B., Meli, R., Filippi, L., Grisanti, S., Massa, F., *et al.*: Cuneus/precuneus as a central hub for brain functional connectivity of mild cognitive impairment in idiopathic REM sleep behavior patients. European Journal of Nuclear Medicine and Molecular Imaging **48**, 2834–2845 (2021)

[3] Lövdal, S., Carli, G., Orso, B., Biehl, M., Arnaldi, D., Mattioli, P., Janzen, A., Sittig, E., Morbelli, S., Booij, J., *et al.*: Investigating the aspect of asymmetry in brain-first versus body-first Parkinson's disease. npj Parkinson's Disease **10**(1), 74 (2024)

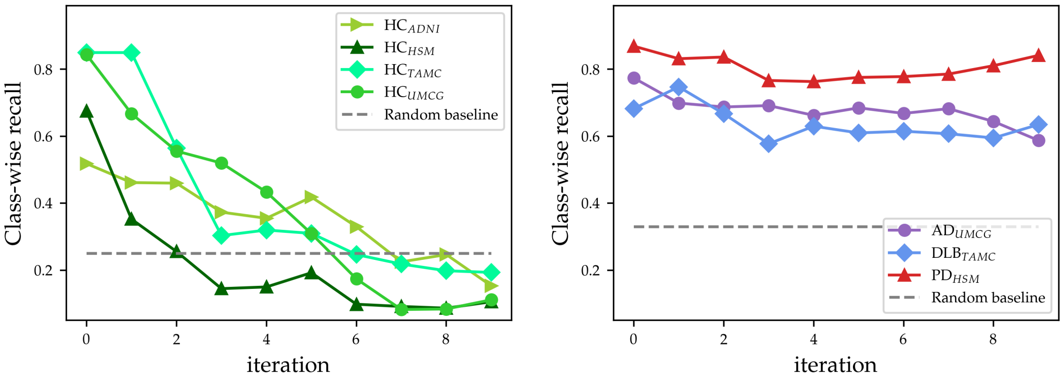

(a) (b)

Fig. 1: (a) Class-specific recall per iteration of IRMA for classifying the center origin of HC subjects of the four centers, as well as disease classification problem (b). Each value represents the mean recall from each of the 100 folds in the 10 times repeated 10-fold cross-validation.

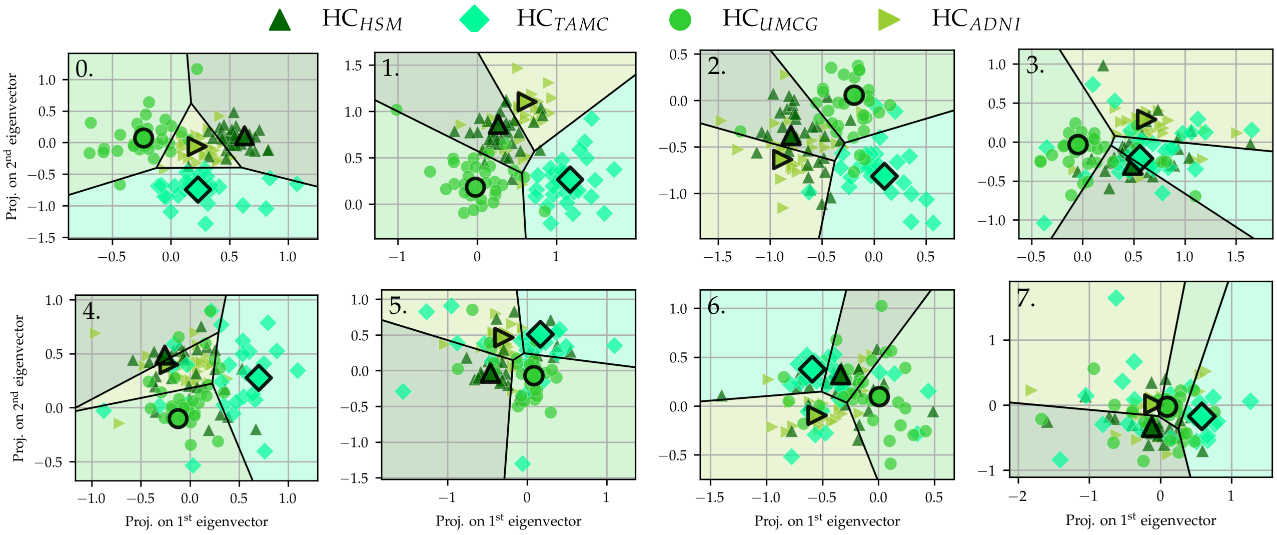

Fig. 2: The full set of HC projected onto the two leading eigenvectors of Λ after training, for each iteration of IRMA. A moderate separation between the groups can still be seen at iteration six. This is estimated (by the cross-validation procedure) to represent overfitting of the model rather than real center-specific signal.

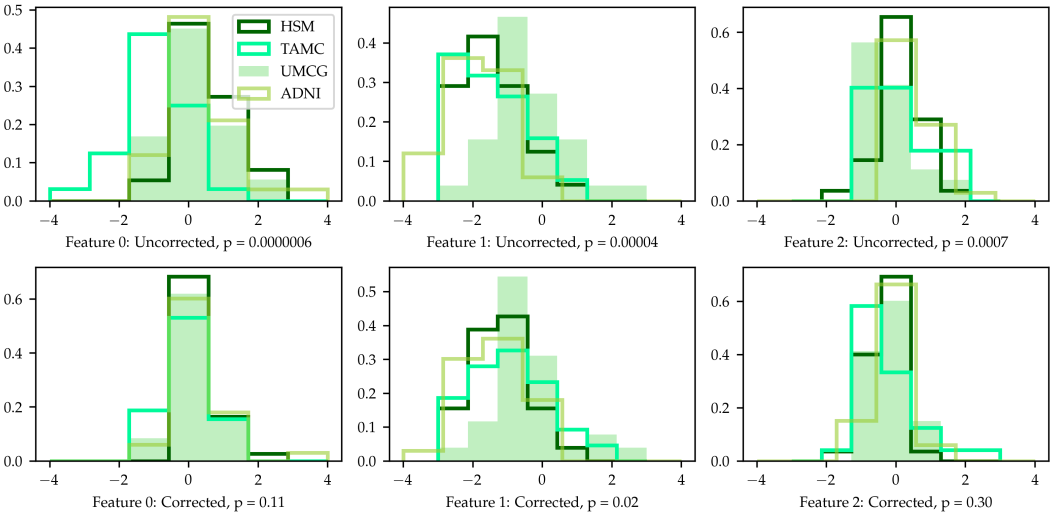

Fig. 3: Example of feature distributions before and after correction, for the three first features being statistically different between the HC cohorts before correction.

[4] Teune, L., Renken, R., De Jong, B., Willemsen, A., Van Osch, M., Roerdink, J., Dierckx, R., Leenders, K.:

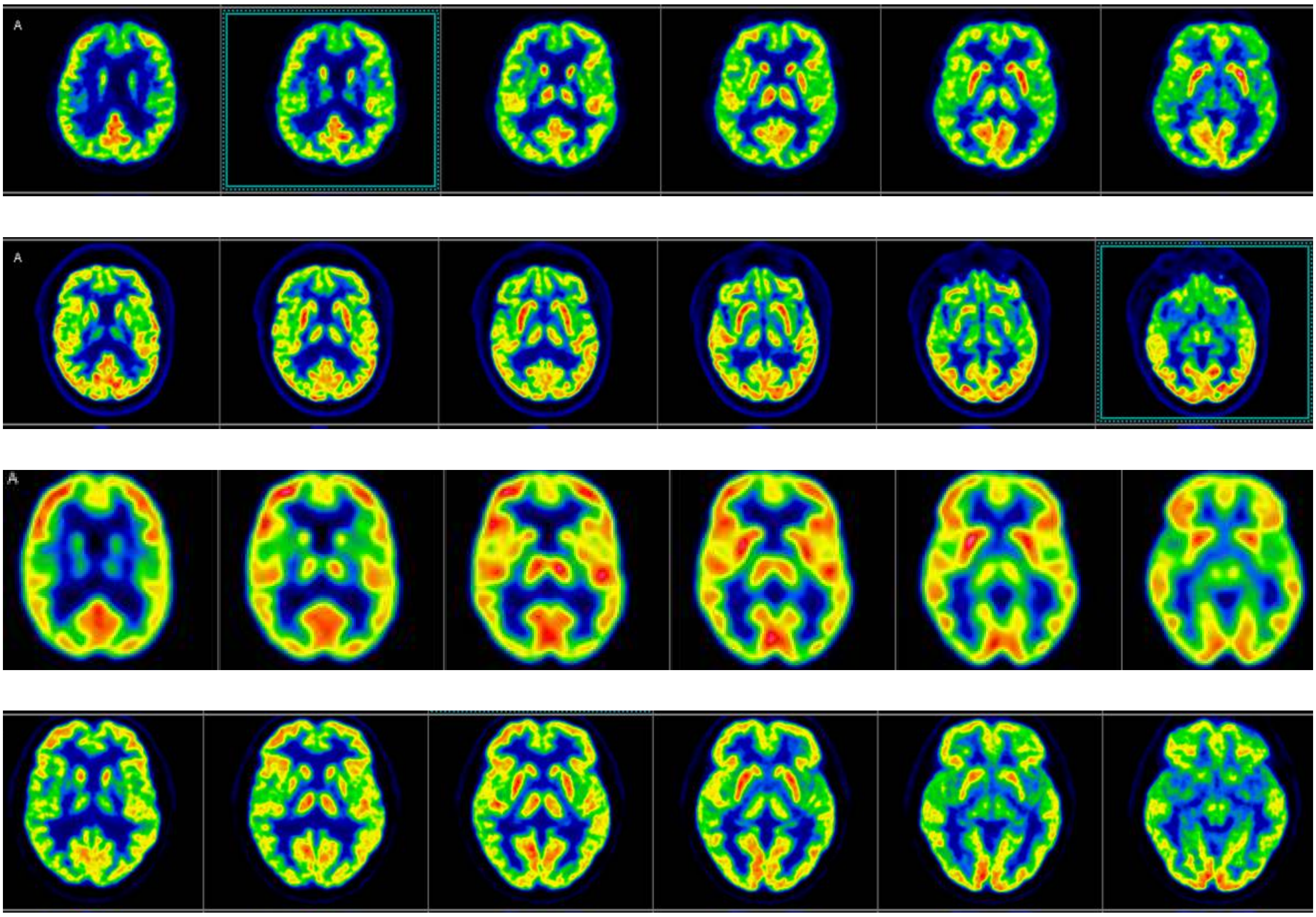

Fig. 4: Example of the image quality of each included camera, as illustrated by the raw scan of an arbitrarily selected control from each center. From top to bottom: HSM, TAMC, UMCG and ADNI. Note that the UMCG images were smoothed on-camera, while the other centers were smoothed during the post-processing and are displayed raw in this image.

Parkinson's disease-related perfusion and glucose metabolic brain patterns identified with PCASL-MRI and FDG-PET imaging. NeuroImage: Clinical **5**, 240–244 (2014)

[5] Meles, S.K., Vadasz, D., Renken, R.J., Sittig-Wiegand, E., Mayer, G., Depboylu, C., Reetz, K., Overeem, S., Pijpers, A., Reesink, F.E., *et al.*: FDG PET, dopamine transporter SPECT, and olfaction: combining biomarkers in REM sleep behavior disorder. Movement Disorders **32**(10), 1482–1486 (2017)

[6] Meles, S.K., Kok, J.G., De Jong, B.M., Renken, R.J., De Vries, J.J., Spikman, J.M., Ziengs, A.L., Willemsen, A.T., Horn, H.J., Leenders, K.L., *et al.*: The cerebral metabolic topography of spinocerebellar ataxia type 3. NeuroImage: Clinical **19**, 90–97 (2018)

[7] Peretti, D.E., Renken, R.J., Reesink, F.E., De Jong, B.M., De Deyn, P.P., Dierckx, R.A., Doorduin, J., Boellaard, R., García, D.V.: Feasibility of pharmacokinetic parametric PET images in scaled subprofile modelling using principal component analysis. NeuroImage: Clinical **30**, 102625 (2021)

[8] Van Veen, R., Meles, S.K., Renken, R.J., Reesink, F.E., Oertel, W.H., Janzen, A., De Vries, G.-J., Leenders, K.L., Biehl, M.: FDG-PET combined with Learning Vector Quantization allows classification of neurodegenerative diseases and reveals the trajectory of idiopathic REM sleep behavior disorder. Computer Methods and Programs in Biomedicine **225**, 107042 (2022)

[9] Petersen, R.C., Aisen, P.S., Beckett, L.A., Donohue, M.C., Gamst, A.C., Harvey, D.J., Jack Jr, C., Jagust, W.J., Shaw, L.M., Toga, A.W., *et al.*: Alzheimer's disease neuroimaging initiative (adni) clinical characterization. Neurology **74**(3), 201–209 (2010)
